# A framework for assessing and managing dependencies in corporate transition plans

**DOI:** 10.1016/j.isci.2025.112811

**Published:** 2025-06-05

**Authors:** Adrien Rose, Gireesh Shrimali, Krista Halttunen

**Affiliations:** 1 Oxford Sustainable Finance Programme, Smith School of Enterprise and the Environment, University of Oxford, OX1 3QY Oxford, UK

**Keywords:** Environmental policy, Social sciences, Business

## Abstract

The urgency of mitigating climate change has increasingly driven companies to develop corporate climate transition plans (CTPs). Factors beyond the direct control of a company can significantly influence the successful implementation of CTPs, but this issue is not addressed comprehensively by existing scientific literature or CTP assessment frameworks. This article introduces the concept of transition plan dependencies, highlighting the necessity of considering external factors such as economic trends, technological advancements, policy environments, and sectoral transitions. Through a combination of a systematic literature review and semi-structured interviews, we propose frameworks and metrics for identifying, quantifying, and managing these dependencies. We use sectoral examples to illustrate the framework and quantification methods, and we suggest next steps to improve the analysis and the management of dependencies in corporate transition plans. This article aims to serve as a foundation for further academic research on transition plan dependencies and their practical applications.

## Highlights

• Companies’ climate plans can be constrained by factors outside their direct control
• Dependencies vary by firms’ characteristics, sectors, and geographies, and over time
• Dependencies can be prioritized using quantitative and qualitative approaches

## Graphical abstract

**Figure:**
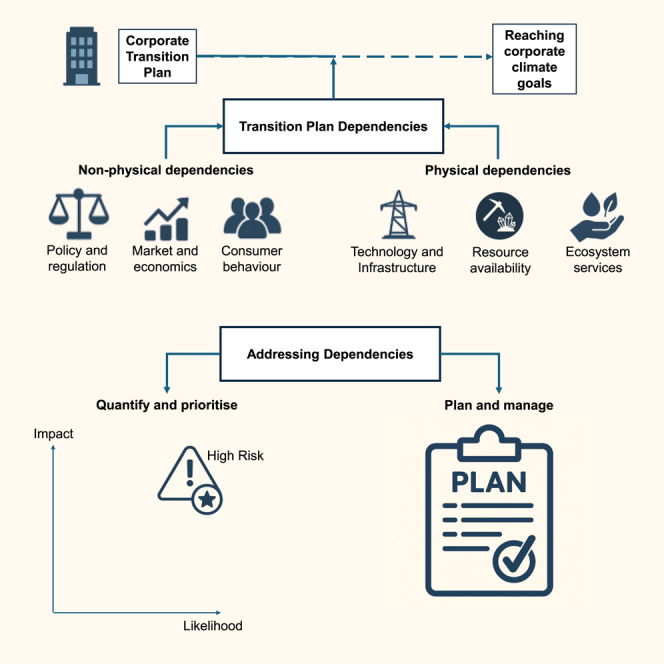

## Introduction

The growing concern about climate change and the need to rapidly reduce global greenhouse gas emissions have given rise to the field of corporate transition planning. Stakeholder and regulator demand around the world is pushing companies to devise their own plans for reducing emissions and participating in the green transition.

Corporate transition plans represent a recent phenomenon that provides a template for companies to disclose strategies and concrete actions to achieve those commitments. These plans provide information on emissions, technology, and investment pathways that an entity plans to follow. There has been an increase in voluntary and mandatory disclosure standards for corporate transition plans, with the publication of the Transition Plan Taskforce Framework, of ISSB’s IFRS S1 and S2, and the European Commission’s Corporate Sustainability Reporting Directive (CSRD) delegated acts. However, corporate climate disclosures remain limited in terms of coverage of disclosing entities and of comprehensiveness and quality of the disclosed information.^1^^,^^2^^,^^3^

This leads to a growing field of assessing the credibility of corporate transition plans (CTPs) with increasing levels of standardization. Transition plans are usually assessed as independent documents, implicitly assuming that the credibility of the plan depends on elements that are internal to it. However, just as a company’s business strategy depends on external factors, the successful implementation of a CTP depends on elements at least partially outside the control of the company that made the plan. These include economic trends, technological breakthroughs, the policy environment, and the pace of the transition in other sectors and among competitors. For instance, Origin Energy postponed the closure of the Eraring power station – Australia’s largest coal-fired power plant – by two years, in response to the New South Wales (NSW) government’s concerns around energy supply.

We call these elements transition plan dependencies. While dependencies clearly impact the credibility of a CTP, most existing frameworks do not include guidance on how to address them (The frameworks reviewed are described in the supplemental information, Appendix S1). We also know of no academic research on the topic. Colesanti Senni et al.,^4^ reviewed disclosure frameworks and elicited experts’ insights to identify sixty-four indicators for assessing CTPs. Four of these indicators focus on a company’s engagement strategy with policymakers, industry associations, and its value chain – i.e., actions to address transition plan dependencies. However, only one indicator focuses on an external factor impacting the success of the corporate transition plans.

To address this gap, the aim of this perspective article is to introduce the concept of dependencies into the academic discourse around the sustainability transition. We explain the concept of dependencies and suggest novel frameworks and metrics as well as future research directions to better manage them when preparing and assessing transition plans. We hope that the ideas presented here act as a useful basis for further work on this under-researched topic.

In this article, we focus primarily on external transition plan dependencies. We define *transition plan dependencies* as physical or non-physical factors on which a company depends to successfully implement its CTP, and *external transition plan dependencies* as transition plan dependencies over which a company has reduced control. Therefore, we focus on threats that are specific to the success of the CTP and not on threats to the overall business strategy of the company, acknowledging that these two concepts often overlap.

## Results and discussion

### Barriers to transition and current practices around transition plan dependencies

#### Barriers to transition and dependencies

**Figure 1:**
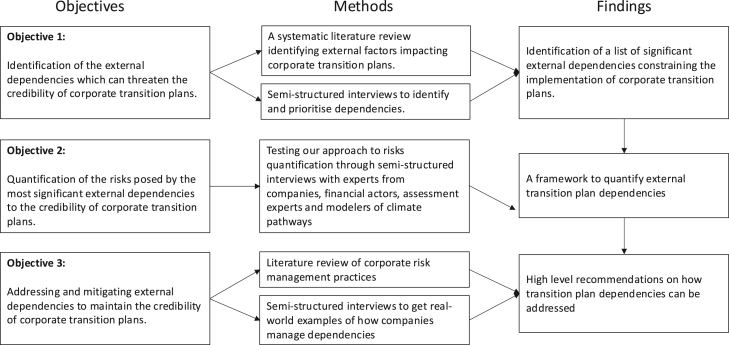
Approach to prioritize and quantify dependencies

There is abundant literature on the barriers that can hinder the transition to a low carbon economy. Depending on company characteristics, these barriers can be outside the direct control of a company and consist of external dependencies that undermine the implementation of a CTP. To highlight why these barriers can threaten the credibility of a CTP, we conducted a systematic literature review of factors that can constrain the deployment of low-carbon solutions (see the section STAR Methods and Figure 1 for the methodology and Table S3 in the supplemental information for a comprehensive summary).

We first identify a series of physical factors. Infrastructure and value chains^5^^,^^6^^,^^7^^,^^8^^,^^9^^,^^10^; technology development^11^^,^^12^^,^^13^^,^^14^; and resource availability^11^^,^^15^^,^^16^^,^^17^^,^^18^^,^^19^ all play a crucial role in scaling climate solutions. For instance, producing low carbon steel can require access to low carbon hydrogen and carbon capture and storage infrastructures, technological breakthroughs in ironmaking and steelmaking, and access to high-grade iron ore. Scaling electric vehicles requires the deployment of charging stations, technological improvements in batteries, and access to critical minerals.

In addition, dependency on ecosystem services^20^^,^^21^ can impact the implementation of low-carbon solutions such as scaling of hydropower in increasingly water-stressed areas. Finally, shortages of skilled professionals with expertise in sustainable practices can also make it difficult for the company to effectively implement and manage its climate transition initiatives.^22^^,^^23^

Through our literature review, we also identify a range of non-physical barriers to decarbonizing the economy. Policies can have wide-ranging impacts to support or hinder corporate mitigation efforts.^24^^,^^25^ Beyond the regulatory framework and policy instruments, the overall policy strategy can influence corporate transition plans by setting technology and investment pathways and targets.^26^^,^^27^

Access to capital and the cost of capital can constrain the development of low-carbon solutions.^13^^,^^28^^,^^29^ Renewable energy projects do not have the same investment profiles as fossil fuel assets, with higher upfront costs making the cost of financing a crucial decision criterion for investors.^30^ Energy and commodity prices also impact the relative competitive advantage of low carbon solutions and, in turn, impact the implementation of corporate transition plans.

Finally, a large literature shows that social acceptance can hinder the development of decarbonization levers.^31^^,^^32^ Customer demand and client behaviors are also critical,^33^ and demand-side interventions can significantly reduce GHG emissions.^34^^,^^35^

Overall, the transition literature highlights that decarbonizing the economy requires systemic socio-technical changes involving significant interdependences.^36^^,^^37^ All the barriers listed above can impact corporate transition plans and limit the ability of companies to deliver on their targets. As a result, stakeholders are increasingly calling for corporate transition plans to include a discussion on the assumptions, uncertainties, and external factors that could impact the implementation of the plan.

#### Accounting for dependencies in existing frameworks

Disclosure frameworks and guidelines from international organizations recommend that firms disclose dependencies to some extent (see Table 1). However, they offer limited guidelines on what constitutes a dependency or what indicators to use to account for them. All frameworks prescribe to disclose assumptions underlying the transition plan, which can include assumptions on factors outside the full control of the company. Glasgow Financial Alliance for Net Zero (GFANZ), the Transition Plan Taskforce framework (TPT), and the European Union’s Corporate Sustainability Reporting Directive (CSRD) list examples of dependencies (e.g., changes in regulatory factors, shifts in consumer preferences, or technology development). In addition, the TPT recommends accounting for the time frame over which dependencies occur and the CSRD suggests breaking down dependencies per decarbonization levers and geographies.

**Table 1:** Existing disclosure guidelines on external dependencies

Organisation	Guidelines
Task Force on Climate-Related Financial Disclosures (TCFD)	Describe the assumptions, uncertainties, and challenges to implement the CTP
Transition Plan Taskforce (TPT)	Disclose the assumptions used in the CTP and the external factors on which the CTP depends, their time frames and their implications for the CTP and for the entity’s financial statements
International Sustainability Standards Board (ISSB)	Disclose assumptions used in the CTP and external factors on which the CTP depends
The European Union’s Corporate Sustainability Reporting Directive (CSRD)	Describe how the CTP depends on the availability and allocation of resources and how future environmental-, societal-, technology-, market- and policy-related developments can impact the implementation of the CTP
Glasgow Financial Alliance for Net Zero (GFANZ) through its report *Expectations for real economy transition plans*	Describe the key assumptions underlying the company’s transition-related business, financial, and operational plans, their implications for the entity’s financial statements, and the impact on the CTP if certain assumptions prove incorrect

*Sources*:^38^^,^^39^^,^^40^^,^^41^^,^^42^^,^^43^^,^^44^^,^^45^^,^^46^^,^^47^^,^^48^^,^^49^^,^^50^. A more detailed version of this table is available in the supplemental information (Table S1) (The table leaves out issues related to the resilience of a company’s business strategy to climate risks as we focus on risks that are specific to the transition plan (though there is overlap between the two issues)).

While disclosure frameworks account for dependencies to some extent, most assessment methodologies do not account for dependencies when rating corporate transition plans (Assessment methodologies reviewed are listed in the supplemental information in the Appendix S1. ATP Col’s framework is an exception, though the methodology directly implements the approach proposed in a preprint version of this article. These methodologies often mention engagement with a company’s value chain to capture a company’s effort to engage with its suppliers and customers. However, most methodologies do not comprehensively capture to what extent the company’s plan relies on external factors^51^^,^^52^^,^^53^).

#### Managing corporate risks and dependencies

While the academic literature focusing on corporate transition plan dependencies is limited, there is extensive literature on managing corporate risk and dependencies that can inform our work.

Existing literature on corporate risk management can be applied to addressing dependencies and the risks they pose to corporate transition plans. The concept of Enterprise-wide Risk Management (ERM)^54^ is particularly relevant to this as it is a systematic approach to the management of the total risks a company faces.^55^ ERM’s core concept is to shift corporate risk management from a silo-based and fragmented approach to a comprehensive approach.^56^ As a result, ERM aims to align a company’s strategy, processes, technologies, and skills to mitigate uncertainties. This is well suited to the risks posed by transition plan dependencies, given the multiple possible sources of dependencies and their interconnectedness. Better risk governance requires involving several hierarchical levels of the company’s structure^57^ and training individuals to be able to identify, understand, and mitigate these risks, which, in the context of climate transition plans, can be new and less known.^58^

Managers’ subjectivity and preferences often affect the measurement, assessment, and mitigation of risks,^54^ making tools which allow actors to identify and prioritize transition plan dependencies objectively valuable. Stress testing,^59^ materiality analysis,^60^ sensitivity analysis,^61^ and scenario analysis^62^ can bring objectivity when assessing what the most critical dependencies are threatening the implementation of a CTP. To quantify the risks associated with dependencies, several approaches can be used. Boholm^63^ states that risk is often defined as the likelihood of a negative event multiplied by its impact, but that less quantitative approaches are common and can be useful, such as classifying risks as high or low. Hargreaves & Mikes^64^ offer four different approaches to risk quantification: using C-suite expertise to identify and manage the largest risks, classifying risks as low, medium, or high according to two dimensions (impact x likelihood), a similar approach to the second one but basing the classification on a company-wide consultation, a statistical analysis which accounts for the fact that risk can materialize partly. We build on this literature to develop our approach to quantifying dependencies in the section on the quantification of dependencies.

Managing dependencies is not specific to CTPs, and there is abundant literature on how companies can address external factors and quantify risks. According to Kotter,^65^ companies undertake several actions to actively manage these dependencies.

(1) Shift to activities and/or geographies where dependencies are less likely to affect the company e.g., prioritize decarbonization levers relying on more mature technologies,
(2) Actively manage dependencies by developing external linkage and control on who works in the domain and how (e.g., develop a joint venture with a company developing carbon capture and storage (CCS), engage with trade associations, and lobby to influence regulation),
(3) Develop a governance structure able to adapt to and anticipate dependencies.

This literature feeds into our discussions on how companies can address dependencies in the section on addressing dependencies.

### Defining and quantifying dependencies

Based on a literature review and semi-structured interviews with transition experts, we propose a typology of dependencies in CTPs as well as a novel approach to quantifying dependencies.

#### Categorization of dependencies

Corporate climate transition plans hinge on a complex network of dependencies, affecting their feasibility, financial viability, and successful implementation. These factors can affect companies directly or throughout their supply chain. The first novel contribution of this article is to provide a structured theoretical framework to better understand the concept of corporate transition plan dependencies. This typology was co-developed with the European Commission’s Joint Research Centre^66^ and it serves as a preliminary tool to identify these dependencies. We divide dependencies into two categories: Physical, and Non-Physical. The definitive version of this typology is presented in Table 2.

**Table 2:** Typology of dependencies that can influence a corporate transition plan

Category	External dependency	Dependency type
1. Non-Physical	1.1 Policy strategy	- National decarbonization strategy - Geopolitical environment (e.g., threats to energy security, trade of critical resources)
1. Non-Physical	1.2 Regulatory framework and policy instruments	- Real economy regulation (e.g., permitting process) - Carbon pricing mechanisms and subsidies - Financial regulation - Legal framework (e.g., ESG litigation risks)
1. Non-Physical	1.3 Market and Economics	- Capital availability and cost - Energy and commodity prices
1. Non-Physical	1.4 Public acceptance	- Concerns about local effects e.g., “Not in my backyard,” just transition - Just transition (e.g., local impact on employment)
1. Non-Physical	1.5 Consumer and client behavior	- Willingness to reduce demand and/or adapt behaviors - Willingness to pay a green premium
2. Physical	2.1. Infrastructure and logistics	- Availability of infrastructure and logistics for transport, distribution, and storage
2. Physical	2.2 Technology	- Technology readiness levels and innovation - Efficiency improvement - Technology lock-in
2. Physical	2.3 Resource availability	- Availability of land, raw materials, and other inputs
2. Physical	2.4 Environmental impacts and ecosystem services	- Climate change impact (e.g., decreased water availability for power generation)
2. Physical	2.5 Labor availability	- Availability of skilled workers

The typology aspires to be exhaustive and was first developed by conducting a systematic literature review exploring how dependencies can constrain the deployment of low-carbon solutions (see sections barriers to transition and dependencies and STAR Methods). Interviews enriched this typology by giving specific, concrete examples of transition plan dependencies and by commenting on the relevance of each category, potential gaps, and the overall structure. Insights from interviews led to new categories (e.g., 1.5 Consumer and client behaviors from interviewees 4, 8, 9, 10, 11, and 13) and helped to refine existing ones (e.g., distinguishing between 1.1 Policy strategy and 1.2 Regulatory framework and policy instruments from interviewee 4).

Dependencies are interconnected and multidimensional, making these categories non-mutually exclusive. A decarbonization lever can face multiple constraints; for example, interviewee 7 noted difficulties in transitioning mining vehicles to low-carbon energy due to both technology and infrastructure challenges. In addition, dependencies are likely to interact with one another. For instance, interviewee 10 mentioned that consumer demand affects low-carbon product scaling, which policymakers can influence through carbon taxes and regulations on green claims. In addition, it appears difficult to disentangle certain mechanisms or decide a hierarchy between dependencies. This suggests that alternative categorizations might be relevant; our goal is to offer an initial framework to structure the discussions around this concept.

Exposure to transition plan dependencies is context-specific and vary across different geographies. Transition experts suggest analyzing dependencies for specific business areas or geographies rather than companywide. A strong enabling environment is the result of factors that vary across geographies such as the policy strategy and regulatory frameworks, economic conditions (e.g., cost of capital being a stronger constraint in some low and middle-income countries), the industrial landscape (e.g., collaboration between actors to develop CCS infrastructure), the resource availability and the competition between actors to secure them, and the geopolitical context.

Firm-specific characteristics influence the ability to address dependencies. This was an essential addition from interviews. Sectoral differences are critical, with decarbonization in sectors such as steel relying on less mature technologies and on the decarbonization of other sectors. A company’s position in the value chain and its degree of vertical integration also affects its control over certain factors. Additionally, a firm’s size and market power impact its ability to influence suppliers and clients. Finally, ownership structure plays a role, with state-owned companies potentially facing fewer constraints on capital and resource availability, as noted by interviewee 11.

Shareholders can play a key role in pushing for an ambitious decarbonization strategy or constraining its implementation. While our framework does not explicitly mention shareholders, this topic came up spontaneously in almost all interviews with participants from real-economy firms. Shareholders could be seen as either an internal or external constraint, depending on the participants.

The return profile of decarbonization solutions, which is affected by dependencies such as 1.3 Market and Economics, appears to be a key determinant of the support or opposition of shareholders to the CTP. This was stressed by interviewees 6, 9, and 13. Comparing the relative attractiveness of the risk profiles of emissive products and low-carbon alternatives is not within the scope of the article. However, low-carbon solutions will be a more credible business prospect if they are profitable and offer higher returns with attractive timelines and risk profiles.

Finally, acknowledging the significance of external dependencies should not prevent actors from taking actions. Interview participants 1 and 2 emphasized the importance of not allowing companies to use this concept to avoid decisive action on the transition. The purpose of the framework is not to delay action but to better understand transition plan credibility and levers for change by identifying relevant dependencies. In the section addressing dependencies, we offer a discussion on the degree of control that companies have over dependencies and how companies can manage them.

#### Quantification of dependencies

The second novel contribution of this study is to propose an approach for quantifying dependencies in CTPs in terms of their significance to the overall credibility of the plan.

##### Approach and metrics

**Figure 2:**
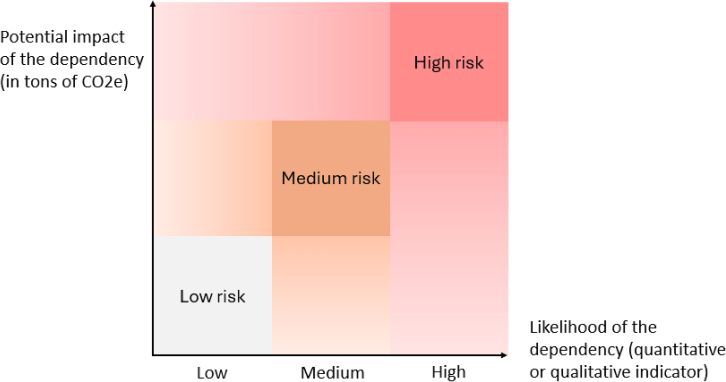
Research design

Our framework recommends evaluating dependencies primarily based on the size of their impact on a transition plan and their likelihood of hindering the transition. We propose metrics for emission reduction at risk (as tons of carbon dioxide equivalent), probability of a dependency preventing planned emission reduction, and the risk of dependency, which is the product of the earlier two metrics. The metrics are summarized in Table 3 and Figure 2 illustrates our approach.

**Table 3:** Proposed metrics for prioritizing CTP dependencies

Object of measurement	Metric	Description
Impact of dependency	Emission reduction at risk (per year/overall)	Total size of the planned reduction in kgCO_2_eq that is at risk of not occurring because of the specific dependency
Probability of dependency	Probability of dependency preventing planned emission reduction (by 2030/2050)	Probability between 0 and 100%, either estimated or calculated
Risk of dependency	Severity of the dependency for a corporate transition plan	Combined metric of impact multiplied by probability of the dependency

Quantifying the impact of a dependency is the most straightforward metric, as supported by most interviewees (see y axis on Figure 2). Corporate transition plans increasingly include quantitative assessments of each decarbonization lever’s contribution to targets, as recommended by the TPT Framework and required by the CSRD in the EU. The expected contribution of a decarbonization lever that relies on an external factor can be used to obtain an upper bound of the potential impact of a dependency. Refining this estimate is possible by having a granular quantitative breakdown of contributions per lever, by isolating specific levers that are affected by a dependency. However, a dependency might only partly materialise and/or partly constrain the implementation of a decarbonization lever, as mentioned by several interviewees.

Scenario analysis can provide estimates of dependency impacts under different assumptions, narrowing the number of plausible scenarios. Besides, scenario analysis is already commonly used in corporate risk management for other challenges such as climate physical risks or energy and commodity pricing. However, to ensure comparability and credibility, it is important that companies are transparent about the scenarios used and their underlying assumptions, for instance, by aligning with feasibility criteria such as those applied by the EU Climate Advisory Board in the EU context.^67^

Our approach was primarily designed to account for the impact of dependencies on planned emission reductions, a key determinant of CTP credibility, but it can also be applied using other variables of interest. Using cumulative emissions reductions at risk would be the most relevant metric to use to assess adherence to a Paris-Aligned carbon budget.^68^ It also accounts for the impact of a delay in the implementation of a transition plan (e.g., a new technology becomes available but later than expected). Using avoided emissions, reduced emissions, or carbon removals captures the impact of dependencies on positive contributions to decarbonizing the economy, as suggested by interviewees 1, 6, and 7, though estimating these metrics is challenging.

Calculating the probability of a dependency materializing is much harder, according to most interviewees. Historical data are not available for many decarbonization levers, and analyses based on historical trends have struggled to correctly estimate the evolution of decarbonization levers such as solar and wind energy.^69^ Interviewees 3 and 13 mentioned that third-party data from experts can be used to estimate probabilities when available and reliable. For instance, the technology readiness levels published by the International Energy Agency could be used to assign probabilities to the availability of specific technologies. Rather than trying to assign exact probabilities, scenario-based approaches allow companies to explore a range of plausible futures and stress-test their strategies under different conditions. Transition roadmaps can also serve as a valuable tool for finding and quantifying dependencies, particularly when developed by actors with the ability to influence the transition and implement them, such as regulatory bodies. Interviewee 13 said that starting with estimates that are backed by simplistic and transparent assumptions and then iterating on the approach could be a valuable first step.

Given the lack of robust quantitative information, qualitative assessments may be needed and can provide a useful first step. Interviewees 11 and 14 suggested using these to separate between the highest and lowest probabilities. Most corporate interviewees mentioned that individuals in the company would be able to have a qualitative estimate of the likelihood and impact of dependency. In-house expert knowledge can be a great source of information but it is often scattered throughout the company, with interviewees 6, 7, and 9 pointing out that the decarbonization effort results from the coordination of various teams, and with interviewees 9 and 13 stating that someone else in the company would have answered differently questions on dependencies’ identification and quantification. As a result, interviewee 2 recommended organizing an objective consultation process to access various in-house expertise and reduce bias.

Marginal abatement cost curves (MACCs) can also be a useful tool to account for some dependencies (e.g., 1.3 Market and Economics, 2.2 Technology, 2.3 Resource Availability, and so forth), depending on the richness of the model and related assumptions. It provides information on the most likely decarbonization levers – the ones with lower marginal abatement costs. Having different MACCs corresponding to different assumptions about dependencies also allows for capturing how transition plan dependencies affect the financial viability of a company’s transition plan. Besides, it enables stakeholders to challenge assumptions made on different dependencies when the assumptions are disclosed transparently.^70^ However, as mentioned by interviewee 4, this approach should also account for elements specific to geographical dependencies. For instance, even if CCS technologies had a low marginal abatement cost, the proximity to a CCS cluster could constrain their use as a credible decarbonization lever.

Overall, interview participants generally supported the corporate risk approach to dependencies, although most of them raised concerns about the feasibility of calculating probabilities, making the risk metric (third metric) an abstract construct that cannot be applied. In addition, interviewee 2 noted that dependencies’ stringency is time-sensitive, suggesting that analyzing dependencies over different periods would improve the quantification exercise. Further work on estimating the likelihood of dependencies, probably focusing on a subset of dependencies in specific sectors and time horizons, could make the likelihood metric easier to use.

Meanwhile, we recommend prioritizing dependencies based on the size of the assessed impact, as data are more easily available for this indicator, especially in cases where calculating the probability of a dependency is challenging. Then, using a qualitative assessment of the probability, it is possible to identify dependencies that pose a higher risk (See Figure 1). This can then be used to prioritize which dependencies require further work, both in terms of risk assessment and planning.

Overall, this framework aims to capture a company’s exposure to transition plan dependencies. Interviewee 9 also pointed out that the list could include another metric about the extent to which a company is able to influence a certain dependency and diminish its risks. The combination of the exposure to dependencies and the strategy and ability to address them would result in a more precise assessment of the risk posed by these dependencies. We have chosen not to develop metrics capturing the latter, but we further discuss the topic in the section addressing dependencies. Further work on quantifying the degree of control of a company over transition plan dependencies and the quality of its strategy to address them would be valuable.

##### Dependencies in practice: sectoral examples

Interviews with corporate transition experts gave illustrative examples of applying the dependency typology and metrics in different sectors of the economy. Later in discussion, we present two simplified examples of quantifying the impact of transition plan dependencies. It should be noted that, while these examples are inspired by real-life companies from the interviews, all numerical values in these examples are illustrative and do not represent actual data.

Company X, a utility company, primarily generates Scope 1 emissions from its coal-fired power plants. Aiming to reduce its absolute Scope 1 emissions by 70% by 2035, the company plans to retire its two coal-fired power plants in 2035 instead of 2040 to reduce its Scope 1 emissions by 35%. However, the company is currently committed to the government to keep producing electricity with these plants until 2040. This jeopardizes half of the emissions reduction and leads to significantly higher cumulative emissions even if it retires its plants in 2040. Increasing concerns around energy security, just transition, and lack of support for renewable energies from local and national policymakers would increase the probability that the firm cannot retire its plants earlier. Therefore, this dependency poses a high risk to the CTP.

Company Y specializes in commercial real estate loans, with most of its Scope 3 emissions coming from downstream investments. Its portfolio is significant, scattered across its country of operation, and representative of the country’s sector. Thus, Company Y finds the policy strategy, regulatory framework, and policy instruments to decarbonize the building sector as its main transition plan dependency. The company quantifies how various levels of implementation of the national decarbonization would affect its CTP, revealing a potentially high impact on the CTP. Given the country’s strong climate policy record of accomplishment and bipartisan support for retrofitting policies, it seems likely that the country will deliver at least partly on its commitments to decarbonize the building sector. Therefore, the dependency is only rated as medium risk.

These examples illustrate the application of our framework: focusing on the most important decarbonization levers, then identifying dependencies which are likely to affect them, and finally estimating the likelihood of this impact using in-house ability, scientific literature, or other relevant data. Combining these two metrics can then provide an estimate of the risk of exposure to a dependency.

These examples are relatively straightforward, but applying this framework to more complex companies comes with practical issues. Firm-specific characteristics can make quantification harder as firms operating across multiple sectors and jurisdictions face greater exposure to diverse dependencies. For instance, interviewees from two financial firms demonstrated this contrast: one operates within a single country and sector, allowing the quantification of policy impacts on the achievement of corporate targets, while the other operates globally across multiple sectors and describes this task as daunting. As pointed out by participants, calculating risk is labour-intensive and requires a lot of historical data to enable clear conclusions.

The ability of a company to analyze dependencies in its CTP also depends on the overall sophistication and maturity of the company’s transition work. Some companies have already carried out granular quantitative analyses of different decarbonization levers, an important first step to analyzing transition plan dependencies. However, this might not be as equally feasible for all firms, with smaller firms or firms operating in low-income countries facing bigger challenges. Interviewee 4 highlighted that exact dependency quantification needs granular scenario analysis, considering local contexts rather than strictly regional or national boundaries. This requires substantial data, often unavailable in certain sectors or in low- and middle-income countries, as noted by interviewee 12.

#### Addressing dependencies

All interviewees supported that, while CTPs rely on external factors, companies have a degree of control over these transition plan dependencies and are responsible for managing them. In this section, we discuss several approaches that can be used to manage transition plan dependencies.

A critical first step in managing dependencies is to conduct a comprehensive analysis. This involves the identification, quantification, and prioritization of dependencies, allowing companies to design effective mitigation strategies. This should be an iterative process to reflect changes in the external environment and improvements in the company’s expertise in transition planning. For instance, interviewees 6 and 9 both mentioned that if the policy framework of a jurisdiction is relatively less favourable than expected, this may lead to higher capital deployment in another region with more attractive climate policies. Similarly, if a dependency on a specific decarbonization lever is more constraining than expected, the company could consider relying more on another lever. By regularly monitoring and reassessing dependencies, companies can anticipate potential disruptions and adjust their CTPs. This article provides actionable tools to conduct this first step.

Transparent reporting on dependencies and planned responses is another crucial element in addressing dependencies. By flagging risks to the transition plan’s implementation, companies can better manage stakeholders' expectations and be transparent about what will be achieved depending on different states of the world. This requires disclosing the different assumptions underlying each scenario, which is increasingly recommended and/or required by international reporting frameworks and regulations. Highlighting trade-offs between a company’s climate strategy and its financial and other sustainability goals clarifies its direction and adaptability to future scenarios. Reporting on dependencies can improve coordination with other actors, such as policymakers. It also allows assessors of transition plans to challenge the assumptions made by the company, for instance, on its degree of control over certain dependencies.

To be relevant, reporting on dependencies should precisely define the dependency and what action is needed from the company or other actors to overcome it. Vague references to external factors do not convey actionable insights and can undermine confidence in the CTP. For instance, saying that the implementation of the CTP requires more supportive net zero policies without defining what the current constraints are and without outlining clear asks for policymakers is insufficient. However, one interviewee mentioned that reporting on dependencies and assumptions could expose a company to reputational or regulatory challenges, particularly given the elevated level of uncertainty around climate data. There are also incentives to misrepresent the stringency of dependencies: either by minimizing them to increase the confidence of stakeholders that the CTP will be implemented, or by making them seem more constraining to avoid taking responsibility and immediate, ambitious actions.

Finally, companies can directly act to mitigate dependencies across various spheres of influence.^71^ Interviewee 6 mentioned that actions could be taken to address most dependencies, saying that cases where a company had no control were rare. The war in Ukraine, and in general geopolitical crises, are examples of such cases, but even then, companies can design contingency plans to account for and adapt to these external factors. Relevant actions mentioned during the interview process, especially by interviewees 6, 9, and 11, include.

(1) securing long-term contracts,
(2) lobbying for policies to support decarbonization,
(3) collaborating with peers, suppliers, or any other relevant stakeholders,
(4) making contingency plans in case a dependency prevents specific emission reductions.

Industry collaboration to further the development of required policies, technologies, infrastructure, and other operating conditions appears particularly relevant to mitigate transition plan dependencies. Interviewee 13 stated that industry coalitions can alleviate capital investment constraints to develop assets that help multiple actors (recycling facility, low-carbon power generation asset, CCS infrastructure). Such coalitions can also guarantee a higher volume of input and/or demand to a third party (e.g., the operator of a recycling facility), thus providing more financial stability to this operator. Finally, they can also be an efficient body to influence customers’ behaviors. While these coalitions can be a powerful lever for change, they may also introduce challenges, particularly concerning competition law.^72^

Developing external linkages enables companies to gain greater control over dependencies.^65^ Cooperation with other companies facilitates the development of firm-specific competencies and fosters the systemic sociotechnical transformations necessary for scaling climate innovations.^73^^,^^74^ Buyer coalitions, as highlighted by interviewee 4 in the context of CCS clusters, play a crucial role in this process; however, cooperative strategies can extend to a broader range of actors. Interview 11, who works for a company providing financial services in the real estate sector, noted that the low take-up of climate mitigation technologies, such as heat pumps, among their clients was limiting the implementation of the company’s climate strategy. To address this dependency, the company introduced financial products designed to incentivize adoption while forming strategic partnerships with heat pump providers. By leveraging their established brand reputation, they enhanced the visibility of the technology, increased consumer trust, and facilitated access to providers, thereby accelerating market uptake. However, interviewee 11 noted that the impact of this initiative remained limited, as policy changes were ultimately the primary driver of widespread adoption.

Overall, dependencies rely on intertwined factors that evolve quickly and should be managed dynamically. Interviewee 9 mentioned that corporate climate strategies are based on assumptions about the future, such as various technology and investment pathways, but these assumptions can prove incorrect and impact the relative attractiveness of decarbonization levers. Interviewee 9 suggested that flexibility was key to deliver on promised emissions reductions, for instance by switching to different decarbonization levers.

Determining the degree of control of a company over a dependency is challenging and specific to each company, as discussed in the section on the quantification of dependencies. Examples from interviews illustrate that this can be subjective and counterintuitive. Emissions from the use of sold products, sometimes considered as outside of companies’ control, may be manageable as revenue from emissive products can be replaced with low-carbon alternatives, as mentioned by interviewee 7. In addition, airplane and automobile manufacturers can reduce their scope 3 emissions from the use of sold products by making their products more efficient, provided that an increase in sales does not offset this reduction. Another interviewee, working in retail, mentioned that emissions from the commute of customers used to be considered outside of the control of the company but are now addressed by rethinking the implementation of shops in city centers. Finally, control over dependencies linked to Scope 1 emissions is not necessarily greater than that for Scope 3 emissions. For instance, one interviewee faces significant infrastructure and technological constraints in transitioning mining equipment to low-carbon energy (Scope 1), yet has more influence over Scope 3 emissions from product use.

Further work on assessing the degree of control that a firm has over a dependency would be extremely valuable to determine when dependencies are used as an excuse not to take ambitious action and when they constitute genuine challenges that require coordination across multiple actors.

### Conclusion

We introduce the concept of dependencies in transition plans and provide tools and guidance for addressing them in CTP development and assessment. The provided frameworks and quantification tools are supported by expert interviews and demonstrated with company-specific examples.

Our literature review, as well as interviews with corporate and non-corporate experts, highlight that companies face genuine constraints in their efforts to decarbonize. However, a common theme across interviews with non-corporate transition experts is the concern that dependencies are used as an excuse not to transition. This article provides the first framework to account for transition plan dependencies, which can help companies to assess and manage their dependencies with more objectivity and transparency. It can also be used by any stakeholders interested in the credibility of corporate transition plans, such as policymakers, financial actors, ESG data providers, or the civil society, to better understand and challenge companies’ plans around external factors.

The topic of transition plan dependencies is under-researched, requiring further work in three key areas: first, generating more empirical data on dependencies; second, assessing their likelihood of materializing; and third, determining the degree of control a company has over a dependency. A clear understanding of dependencies makes it possible to realistically evaluate transition plans, identify the largest risks, and focus transition work on areas that require the most urgent attention to keep global climate goals within reach.

### Limitations of the study

This article offers an initial conceptual framework for understanding and assessing transition plan dependencies, supported by illustrative examples and expert insights. However, several limitations should be acknowledged. First, the sample of interview participants is skewed toward high-income European countries, which may lead to a Eurocentric framing of the issues discussed. Second, the study serves as a foundational step, leaving several questions open. As a result, we aim to be transparent about the scope and limitations of the article, and we suggest key areas for further research. Finally, the absence of aggregate data on corporate practices related to transition plan dependencies limits our ability to draw generalizable conclusions.

## Resource availability

### Lead contact

Adrien Rose is the lead contact for this article. (adrien.rose@smithschool.ox.ac.uk).

### Materials availability

Beyond the interview transcripts, this study did not generate new materials.

### Data and code availability

• Data availability: The interview transcripts are not publicly available as making them publicly available could expose sensitive or identifiable information. This was discussed with participants and the ethics committee reviewing our study (Ethics reference: SOGE C1A 24 26).
• Code availability: This study did not generate original code.
• Other items: The study did not generate other items.

## Acknowledgments

We would like to express our gratitude to Nicolas Pickard-Garcia, Thomas Gourdon, Alice Martiny, and Isabelle Seigneur, from the European Commission Joint Research Center for their contributions to the categorization of corporate transition plan dependencies. We would also like to thank all the individuals who participated in the interviews and the workshop. Funding for this work was provided by Santander Group through their support for the Transition Finance Center of Excellence. We also acknowledge the input provided by Santander Group and would like to particularly thank Steffen Kram, Christopher Vernon, Christopher Mogridge, Charlie Liechti, and Etienne Butruille for their feedback. The views expressed in this article are solely the responsibility of the authors and do not necessarily reflect the opinions of the acknowledged individuals.

## Author contributions

Conceptualization, A.R., K.H., and G.S.; methodology, K.H.; formal analysis; A.R.; writing – original draft, A.R. and K.H.; writing – review and editing, A.R., K.H., and G.S; funding acquisition, G.S.; project administration; G.S.; supervision; K.H. and G.S.

## Declaration of interests

The authors declare no competing interests.

## Declaration of generative AI and AI-assisted technologies in the writing process

During the preparation of this paper, the authors used ChatGPT and Grammarly to proofread the manuscript’s language and design the graphical abstract. After using these tools, the authors reviewed and edited the content as needed and take full responsibility for the content of the publication. All words included in the final published manuscript were written by the authors.

## STAR★Methods

The discussion in this paper is based on a review of literature and documents giving guidelines for transition-related corporate disclosures and plans as well as ‘systematising expert interviews’.^75^

A systematic literature review was conducted to identify the most common external dependencies in corporate transition plans and the constraints to decarbonisation levers. The literature review was conducted on Google Scholar by searching for all the combinations of the two lists below. The first list was elaborated using the decarbonisation levers prioritised in the International Energy Agency’s Net Zero by 2050 updated report published in 2023: *Low Carbon Solutions* OR *Low Carbon Transitio*n OR *Renewable Energy* OR *Solar Energy* OR *Wind Energy* OR *Battery Storage* OR *Hydropower* OR *Green Hydrogen* OR *Electrolysers* OR *Nuclear Energy* OR *Sustainable Aviation Fuels* OR *Biofuels* OR *Energy Efficiency* OR *Electrification* OR *Electric Vehicle* OR *Heat Pump* OR *Carbon Capture Utilisation and Storage* OR *Carbon Removals*. The second list is designed to capture factors that are likely to constrain the deployment of low-carbon solutions: *External Dependency* OR *Constraints* OR *Contingency* OR *Social Acceptance* OR *Scaling*.

### Experimental model and study participant details

In addition, we carried out a total of 14 interviews with 18 individuals (two interviews had three participants). Five interviews were conducted with general transition experts and nine with sector-specific corporate experts. Table S2 and Appendix S2 in the supplemental information section respectively provide key characteristics on the interviewees and information on the interview method and process. The interviews were used to add richness and context to the developed frameworks and metrics as well as seek feedback and additions to the research approach and outputs.

Interviewees were selected using purposive sampling to ensure each interviewee had relevant expertise.^76^ Non-corporate experts, drawn from academia or standard-setting organizations, were included for their direct focus on corporate transition planning and their more independent perspectives. Corporate experts held senior sustainability roles such as head of sustainability. They handled their companies’ overarching decarbonisation strategy or its implementation within specific business units. These interviewees were selected because their companies published a corporate transition plan which mentioned an external barrier that we would consider a transition plan dependency. Our goal was to gather insights from participants on how they identify, evaluate, and manage these constraints, as well as the processes involved. Overall, our sample includes a higher proportion of participants from high-income European countries, which may result in a more Eurocentric perspective of this issue. Our sample includes 50% of women and 50% of men, though gender is not a focus of our analysis.

### Method details

Thematic analysis was used to interpret the raw output from the semi-structured interviews.^77^^,^^78^ Data were coded and arranged across several themes, the main three being identifying and categorising dependencies, quantifying dependencies, and addressing dependencies. The findings informed the development of the framework and metrics presented in the section quantification of dependencies and added to the sectoral examples that illustrate their application. The research design is summarised in Figure 2.
