# Supplementary material for: A framework for assessing and managing dependencies in corporate transition plans
Source: iScience. 2025 Jun 5;28(7):112811. doi: 10.1016/j.isci.2025.112811 (PMC12256347; doi:10.1016/j.isci.2025.112811)
Supplement: Document S1. Tables S1–S3 and Appendix S1–S3 [file mmc1.pdf]

iScience, Volume *28*

Supplemental information

A framework for assessing and managing dependencies in corporate transition plans

Adrien Rose, Gireesh Shrimali, and Krista Halttunen

Supplemental Information

Table S1. Existing disclosure guidelines on external dependencies (detailed version)

**Organisation**	**Guidelines**
**Task Force on Climate-Related Financial Disclosures (TCFD) through its report *Guidance on metrics, targets, and transition plans***	"The transition plan describes the assumptions, uncertainties, and challenges the organization faces in successfully executing its transition plan."
**Transition Plan Taskforce (TPT) through the *TPT Framework***	Element 1.3 of the framework: "An entity shall disclose:<br>a. the nature of the key assumptions that it uses and external factors on which it depends, and their implications for the achievement of the Strategic Ambition of its transition plan;<br>b. the timeframes over which any key assumptions and external factors under 1.3.a. are expected to occur<br>c. whether and how the key assumptions under 1.3.a. are reflected in the entity's financial statements."
**International Sustainability Standards Board (ISSB) through the *International Financial Reporting Standards (IFRS) S2***	"14.a)iv) (…) Specifically, an entity shall disclose information about: any climate-related transition plan the entity has, including information about key assumptions used in developing its transition plan, and dependencies on which the entity's transition plan relies"
**The European Union's Corporate Sustainability Reporting Directive (CSRD) through the *European Sustainability Reporting Standards (ESRS) S1 and S2***	"The undertaking shall explain if and to what extent its ability to implement the actions depends on the availability and allocation of resources". "Future environmental-, societal-, technology-, market- and policy-related developments" are also mentioned as key elements which can impact the ability to implement the transition plan.
**Glasgow Financial Alliance for Net Zero (GFANZ) through its report *Expectations for real economy transition plans***	The report recommends that firms:<br>• "Describe the key assumptions underlying the company's transition-related business, financial, and operational plans (e.g., reliance on technologies the company is currently not deploying at scale; reliance on actions of its value chain; reliance on specific regulatory policies).<br>• Disclose how these assumptions are reflected in the company's financial statements and audit reports.<br>• Articulate the impact on the transition plan if certain assumptions prove incorrect (e.g. low,

	medium, high impact to achieving net zero), including how much can be achieved now.”

Appendix S1. Assessment methodologies reviewed for Section 2.2

Assessment methodologies reviewed include Accelerate Climate Transition [1], CDP’s assessment conducted using its climate change questionnaire [2] , assessments performed for Climate Action 100+ which include the Net Zero Company Benchmark Disclosure Framework Assessment conducted by the Transition Pathway Initiative Centre (TPI Centre) [3], the Climate Accounting and Audit Assessment conducted by the Climate Tracker Initiative (CTI) [4], the Climate Policy Engagement Alignment conducted by InfluenceMap [5], Capital Allocation Alignment methodologies conducted either by the Climate Tracker Initiative or the Rocky Mountain Institute depending on the sector [6], [7], [8].

The guidance published by the Assessing companies Transition Plans Collective (ATP-Col) [9] mentions the need to account for transition plan dependencies. The authors of this paper are part of the collective and the framework directly builds on this paper, among other resources, to provide recommendations on how to assess transition plan dependencies.

Methodologies developed by commercial assessors of corporate transition plans were not considered as these methodologies are rarely open source or available publicly only to a limited extent.

Table S2. Interviewees' characteristics.

Interview number	Area where the interviewee is based	Country Income Level (World Bank)	Type	Gender	Sector
1	Europe	High-income	Non-corporate	Female	Academia
2	Europe	High-income	Non-corporate	Female	Academia
3	Europe	High-income	Non-corporate	Male	Assessment standard
4	Europe	High-income	Non-corporate	Female	International organisation
5	Europe	High-income	Non-corporate	Male and Female (3 participants)	International organisation
6	Europe	High-income	Corporate	Male	Energy
7	Europe	High-income	Corporate	Female	Industry
8	Europe	High-income	Corporate	Male and Female (3 participants)	Finance
9	Europe	High-income	Corporate	Male	Energy
10	North America	Upper-middle-income	Corporate	Female	Consumer goods
11	Europe	High-income	Corporate	Female	Finance
12	Asia	Lower-middle-income	Corporate	Male	Energy
13	Europe	High-income	Corporate	Female	Consumer goods
14	South America	High-income	Corporate	Male	Aviation

Note: Energy includes power generation and oil and gas. Industry could include mining, steelmaking, cement. Finance includes banking services.

Appendix S2. Interview methodology and process

The premise for the interviews is that experts have special knowledge of facts about the external world – in this case, the external transition plan dependencies. This is an appropriate method for an academic field that is substantively new [10]. However, it is important to keep in mind that experts cannot be treated as sources of objective truth [11]. The purpose of the interviews was to include various viewpoints in the discussion of this paper.

All interviews were carried out online via the Microsoft Teams platform between February and May 2024, lasting between 35 and 60 minutes. The interviews followed a semi-structured format, in which an interview guide had been developed based on literature, but the discussion was allowed to proceed organically around the pre-selected themes. Part of each interview was spent reviewing in-progress versions of the frameworks and metrics presented in the section *Quantification of dependencies* of this paper. For the company expert interviews, the transition plan documents of the

company were reviewed by the interviewers ahead to guide the discussion. In two interviews, several people from the same organisation took part at the same time, so the discussions took the form of small group interviews.

The qualitative nature of our approach implies that we cannot make any generalisation on how experts approach and perceive transition plan dependencies. The broad range of possible interview participants means that it is not possible to define and reach a specific sample size that would allow general conclusions to be drawn so we did not aim to conduct statistical analyses of the interviews' content.

Appendix S3. Keywords used to conduct the systematic literature review

The literature review was conducted on Google Scholar by searching for all the combinations of the two lists below. The first list was elaborated using the decarbonisation levers prioritised in the International Energy Agency's Net Zero by 2050 updated report published in 2023. The second list is designed to capture factors that are likely to constrain the deployment of low-carbon solutions.

List 1: Low Carbon Solutions OR Low Carbon Transition OR Renewable Energy OR Solar Energy OR Wind Energy OR Battery Storage OR Hydropower OR Green Hydrogen OR Electrolysers OR Nuclear Energy OR Sustainable Aviation Fuels OR Biofuels OR Energy Efficiency OR Electrification OR Electric Vehicle OR Heat Pump OR Carbon Capture Utilisation and Storage OR Carbon Removals.

List 2: External Dependency OR Constraints OR Contingency OR Social Acceptance OR Scaling

Table S3. Typology of dependencies that can influence a corporate transition plan and rationale to include each category

**Category**	**External dependency**	**Rationale to include**
**Non-physical**	1. Policy strategy	Beyond the regulatory framework and policy instruments, the overall policy strategy can influence corporate transition plans by setting technology and investment pathways and targets. [12], [13].
	2. Regulatory framework and policy instruments	Policies can have wide-ranging impacts to support or hinder corporate mitigation efforts [14], [15], [16], [17], [18], [19], [20], [21], [22], [23], [24], [25], [26]. Corporate transition plans' implementation could be based on climate policies -current or expected - which end up less supportive and ambitious, face reversals, or support climate solutions that are not included in their plans (e.g. the debate over the inclusion of nuclear energy in the European Green Taxonomy). Obtaining permits is also a key issue in enabling the growth of low-carbon solutions [27].
	3. Market and Economics	Access and cost of capital can constrain the development of low-carbon solutions [19], [28] and financial actors can support

		corporate climate mitigation efforts [29]. Renewable energy projects have different investment profiles than fossil fuel assets, with higher upfront costs making the cost of financing a crucial decision criterion for investors (International Energy Agency, 2023b).<br>Energy and commodity prices will impact the implementation of corporate transition plans. Higher expected oil prices can make oil investments more profitable and attractive, leading companies to revise part of their transition plans. An increase in prices of certain commodities such as steel and cement can increase the price of renewable energy (International Energy Agency, 2023b) and impact its competitive advantage.
	4. Public acceptance	Social acceptance can also hinder the development of several energy technologies [21], [30], [31], [32], [33], [34], [35], [36], or more broadly mitigation technologies such as CCS (Carbon Capture and Storage) [37], [38], [39], [40], [41]. Adopting a fair approach to the net zero transition will facilitate the acceptability of low-carbon solutions [42].
	5. Consumer and client behaviour	Demand-side interventions can significantly reduce GHG emissions [43], [44], [45], [46], [47]. Consumers' willingness to pay a green premium is also an important determinant of the take up of low-carbon solutions [48].
**Physical**	1. Infrastructure and logistics	Infrastructure and value chains are crucial to scale climate solutions as they can play a uniquely critical enabling role [49]. For instance, deploying electric vehicles cannot happen without the deployment of charging station, scaling solar and wind requires the development of batteries and improvements of the electricity grid [50], [51], [52], decarbonisation strategies in the shipping industry partly rely on changes in port infrastructures, the availability of transport and storage infrastructure can also constrain the potential of CCS [53]. How a recent technology interacts with the existing infrastructure is also important [54]. Accounting for these essential infrastructure changes in the cost curves of low-carbon solutions is crucial to estimate the feasible potential of these solutions [55]. Logistics of the deployment of low-carbon solutions can also be complex and make installations planning difficult [56].
	2. Technology	Technology development plays a significant role in decarbonising and can prove an important constraint in scaling low-carbon solutions (International Energy Agency, 2023a), especially in sectors where significant emissions reductions come from technology which are not commercially available yet such as steel [58]. Relying on technologies with low technology readiness levels can make it more challenging for companies to scale their decarbonisation efforts. Even the potential of technologies that are already commercially available such as for solar energy can be constrained by technological improvements [28], [59]. Technological lock-in can also impact the deployment of low-carbon solutions [60].
	3. Resource availability	Resource availability might constrain the development of low-carbon solutions at a large scale [61], [62], [63], [64]. According to the World Energy Outlook, global production of lithium, nickel, and

		copper in 2030 will only reach respectively 60%, 80%, and 85% of the global needs in the IEA NZE scenario [65]. Another example is the availability of high-grade iron ore which is required for DRI in steelmaking a production method that is key in transitioning the steel industry to net zero [66]. Critical resources for the transition are not spread evenly among the world, with Indonesia expected to account for 90% of planned nickel refining facilities in 2030 or China accounting for half of planned lithium chemical plants [65]. International relationships and reliable supply chains are likely to impact companies' procurement strategies. Land availability and competition between uses are also a key concern which could constrain low-carbon solutions [23], [67], [68], [69], [70], [71].
	4. Environmental impacts and ecosystem services	Dependency on ecosystem services [72] can impact the implementation of low-carbon solutions such as scaling of hydropower in increasingly water-stressed areas. Besides, climate change impacts on these ecosystem services can reduce the potential of renewable energy [73].
	5. Labour availability	Shortages of skilled professionals with expertise in sustainable practices can make it difficult for the company to effectively implement and manage its climate transition initiatives [19], [74]. For instance, the UK Commission for Employment and Skills highlights shortages of technicians specialising in renewable energy, especially wind turbines, and nuclear energy while increased investment and deployment targets lead to higher workforce needs [75].

References used in the supplemental information

[1] ACT, 'Sector Methodology, Generic', Apr. 2021. Accessed: Dec. 04, 2023. [Online]. Available: https://actinitiative.org/wp-content/uploads/pdf/act-generic-methodology.pdf

[2] CDP, 'Climate Change 2023 Questionnaire', 2023. Accessed: May 26, 2023. [Online]. Available: https://guidance.cdp.net/en/guidance?cid=46&ctype=theme&idtype=ThemeID&incchild=1&microsite=0&otype=Questionnaire&tags=TAG-585%2CTAG-605%2CTAG-599%2CTAG-13145%2CTAG-13135%2CTAG-13140

[3] Climate Action 100+, 'Climate Action 100+ Net Zero Company Benchmark Disclosure Framework Assessment Methodology', Oct. 2023. Accessed: Dec. 05, 2023. [Online]. Available: https://www.climateaction100.org/wp-content/uploads/2023/10/CA100-Benchmark-2.0-Disclosure-Framework-Methodology-Confidential-October-2023.pdf

[4] Climate Tracker Initiative, 'CARBON TRACKER METHODOLOGIES Climate Action 100+ Net Zero Company Benchmark: Climate Accounting and Audit Assessment', Oct. 2023.

[5] InfluenceMap, 'InfluenceMap's Climate Policy Engagement Alignment Assessments: Methodology', Sep. 2023. Accessed: Dec. 05, 2023. [Online]. Available: https://www.climateaction100.org/wp-content/uploads/2023/10/2023-InfluenceMap-Methodology.pdf

[6] Carbon Tracker Initiative, 'CARBON TRACKER METHODOLOGIES Electric Utilities', Aug. 2023. Accessed: Dec. 05, 2023. [Online]. Available: https://www.climateaction100.org/wp-content/uploads/2023/10/2023-CTI-Electric-Utility-Methodology.pdf

[7] Carbon Tracker Initiative, 'CARBON TRACKER METHODOLOGIES Oil and Gas Companies', Aug. 2023. Accessed: Dec. 05, 2023. [Online]. Available: https://www.climateaction100.org/wp-content/uploads/2023/10/2023-CTI-Oil-and-Gas-Methodology.pdf

[8] Rocky Mountain Institute, 'Climate Action 100+ Net Zero Benchmark Methodologies and metrics for assessing focus company capital alignment', Sep. 2023. Accessed: Dec. 05, 2023. [Online]. Available: https://www.climateaction100.org/wp-content/uploads/2023/10/2023-RMI-Methodology.pdf

[9] R. Poivet and A.-C. Assessing Transition Plan Collective, 'Assessing the credibility of a company's transition plan: framework and guidance', Jun. 2024. Accessed: Jul. 11, 2024. [Online]. Available: https://assets.worldbenchmarkingalliance.org/app/uploads/2024/06/Guidance-on-assessing-Companies-Transition-plans_Public-consultation-3.pdf

[10] U. Flick, *The SAGE Handbook of Qualitative Data Collection*. 2018. doi: 10.4135/9781526416070.

[11] A. Bogner and W. Menz, 'The Theory-Generating Expert Interview: Epistemological Interest, Forms of Knowledge, Interaction', in *Interviewing Experts*, London: Palgrave Macmillan UK, 2009, pp. 43–80. doi: 10.1057/9780230244276_3.

[12] S. Eskander, C. Higham, M. Hamley, J. Setzer, and S. Fankhauser, 'Testing the Ambition Loop: Do Country- and Company-Level Net-Zero Targets Reinforce Each Other? A Global Comparison', *Journal of Comparative Policy Analysis: Research and Practice*, pp. 1–17, Apr. 2024, doi: 10.1080/13876988.2024.2317949.

[13] K. S. Rogge and K. Reichardt, 'Policy mixes for sustainability transitions: An extended concept and framework for analysis', *Res Policy*, vol. 45, no. 8, pp. 1620–1635, Oct. 2016, doi: 10.1016/j.respol.2016.04.004.

[14] Q. Abbas *et al.*, ‘Scaling up renewable energy in Africa: measuring wind energy through econometric approach’, *Environmental Science and Pollution Research*, vol. 27, no. 29, pp. 36282–36294, Oct. 2020, doi: 10.1007/s11356-020-09596-1.

[15] O. Bayulgen and J. W. Ladewig, ‘Vetoing the future: political constraints and renewable energy’, *Env Polit*, vol. 26, no. 1, pp. 49–70, Jan. 2017, doi: 10.1080/09644016.2016.1223189.

[16] J. Hou, P. Zhang, Y. Tian, X. Yuan, and Y. Yang, ‘Developing low-carbon economy: Actions, challenges and solutions for energy savings in China’, *Renew Energy*, vol. 36, no. 11, pp. 3037–3042, Nov. 2011, doi: 10.1016/j.renene.2011.03.033.

[17] M. Kittel and W.-P. Schill, ‘Renewable energy targets and unintended storage cycling: Implications for energy modeling’, *iScience*, vol. 25, no. 4, p. 104002, Apr. 2022, doi: 10.1016/j.isci.2022.104002.

[18] J. Li and J. Huang, ‘The expansion of China’s solar energy: Challenges and policy options’, *Renewable and Sustainable Energy Reviews*, vol. 132, p. 110002, Oct. 2020, doi: 10.1016/j.rser.2020.110002.

[19] K. D. Patlitzianas, H. Doukas, and J. Psarras, ‘Enhancing renewable energy in the Arab States of the Gulf: Constraints & efforts’, *Energy Policy*, vol. 34, no. 18, pp. 3719–3726, Dec. 2006, doi: 10.1016/j.enpol.2005.08.018.

[20] E. A. Pina, M. A. Lozano, and L. M. Serra, ‘Assessing the influence of legal constraints on the integration of renewable energy technologies in polygeneration systems for buildings’, *Renewable and Sustainable Energy Reviews*, vol. 149, p. 111382, Oct. 2021, doi: 10.1016/j.rser.2021.111382.

[21] P. Polinori, ‘Wind energy deployment in wind farm aging context. Appraising an onshore wind farm enlargement project: A contingent valuation study in the Center of Italy’, *Energy Econ*, vol. 79, pp. 206–220, Mar. 2019, doi: 10.1016/j.eneco.2019.04.002.

[22] K. S. Rogge and P. Johnstone, ‘Exploring the role of phase-out policies for low-carbon energy transitions: The case of the German Energiewende’, *Energy Res Soc Sci*, vol. 33, pp. 128–137, Nov. 2017, doi: 10.1016/j.erss.2017.10.004.

[23] B. M. Smyth, B. P. Ó Gallachóir, N. E. Korres, and J. D. Murphy, ‘Can we meet targets for biofuels and renewable energy in transport given the constraints imposed by policy in agriculture and energy?’, *J Clean Prod*, vol. 18, no. 16–17, pp. 1671–1685, Nov. 2010, doi: 10.1016/j.jclepro.2010.06.027.

[24] G. R. Timilsina and K. U. Shah, 'Filling the gaps: Policy supports and interventions for scaling up renewable energy development in Small Island Developing States', *Energy Policy*, vol. 98, pp. 653–662, Nov. 2016, doi: 10.1016/j.enpol.2016.02.028.

[25] M. Wang and J. Zhao, 'Are renewable energy policies climate friendly? The role of capacity constraints and market power', *J Environ Econ Manage*, vol. 90, pp. 41–60, Jul. 2018, doi: 10.1016/j.jeem.2018.05.003.

[26] S. Zhou, Y. Wang, Y. Zhou, L. E. Clarke, and J. A. Edmonds, 'Roles of wind and solar energy in China's power sector: Implications of intermittency constraints', *Appl Energy*, vol. 213, pp. 22–30, Mar. 2018, doi: 10.1016/j.apenergy.2018.01.025.

[27] International Energy Agency, 'World Energy Investment 2023', May 2023, Accessed: Nov. 28, 2023. [Online]. Available: https://www.iea.org/reports/world-energy-investment-2023

[28] E. Kabir, P. Kumar, S. Kumar, A. A. Adelodun, and K.-H. Kim, 'Solar energy: Potential and future prospects', *Renewable and Sustainable Energy Reviews*, vol. 82, pp. 894–900, Feb. 2018, doi: 10.1016/j.rser.2017.09.094.

[29] X. Zhao, Y. Xue, and L. Ding, 'Implementation of low carbon industrial symbiosis systems under financial constraint and environmental regulations: An evolutionary game approach', *J Clean Prod*, vol. 277, p. 124289, Dec. 2020, doi: 10.1016/j.jclepro.2020.124289.

[30] M. Segreto *et al.*, 'Trends in Social Acceptance of Renewable Energy Across Europe—A Literature Review', *Int J Environ Res Public Health*, vol. 17, no. 24, p. 9161, Dec. 2020, doi: 10.3390/ijerph17249161.

[31] X. Yuan, J. Zuo, R. Ma, and Y. Wang, 'How would social acceptance affect nuclear power development? A study from China', *J Clean Prod*, vol. 163, pp. 179–186, Oct. 2017, doi: 10.1016/j.jclepro.2015.04.049.

[32] H.-C. Chin, W.-W. Choong, S. R. Wan Alwi, and A. H. Mohammed, 'Issues of social acceptance on biofuel development', *J Clean Prod*, vol. 71, pp. 30–39, May 2014, doi: 10.1016/j.jclepro.2013.12.060.

[33] A. Jobert, P. Laborgne, and S. Mimler, 'Local acceptance of wind energy: Factors of success identified in French and German case studies', *Energy Policy*, vol. 35, no. 5, pp. 2751–2760, May 2007, doi: 10.1016/j.enpol.2006.12.005.

[34] C. W. Klok, A. F. Kirkels, and F. Alkemade, ‘Impacts, procedural processes, and local context: Rethinking the social acceptance of wind energy projects in the Netherlands’, *Energy Res Soc Sci*, vol. 99, p. 103044, May 2023, doi: 10.1016/j.erss.2023.103044.

[35] J. Cousse, ‘Still in love with solar energy? Installation size, affect, and the social acceptance of renewable energy technologies’, *Renewable and Sustainable Energy Reviews*, vol. 145, p. 111107, Jul. 2021, doi: 10.1016/j.rser.2021.111107.

[36] M. Wolsink, ‘The research agenda on social acceptance of distributed generation in smart grids: Renewable as common pool resources’, *Renewable and Sustainable Energy Reviews*, vol. 16, no. 1, pp. 822–835, Jan. 2012, doi: 10.1016/j.rser.2011.09.006.

[37] Federico d’Amore, L. Lovisotto, and F. Bezzo, ‘Introducing social acceptance into the design of CCS supply chains: A case study at a European level’, *J Clean Prod*, vol. 249, p. 119337, Mar. 2020, doi: 10.1016/j.jclepro.2019.119337.

[38] K. van Alphen, Q. van Voorst tot Voorst, M. P. Hekkert, and R. E. H. M. Smits, ‘Societal acceptance of carbon capture and storage technologies’, *Energy Policy*, vol. 35, no. 8, pp. 4368–4380, Aug. 2007, doi: 10.1016/j.enpol.2007.03.006.

[39] B. van der Zwaan, K. Broecks, and F. Dalla Longa, ‘Deployment of CO2 capture and storage in Europe under limited public acceptance—An energy system perspective’, *Environ Innov Soc Transit*, vol. 45, pp. 200–213, Dec. 2022, doi: 10.1016/j.eist.2022.10.004.

[40] N. M. A. Huijts, C. J. H. Midden, and A. L. Meijnders, ‘Social acceptance of carbon dioxide storage’, *Energy Policy*, vol. 35, no. 5, pp. 2780–2789, May 2007, doi: 10.1016/j.enpol.2006.12.007.

[41] N. Markusson *et al.*, ‘A socio-technical framework for assessing the viability of carbon capture and storage technology’, *Technol Forecast Soc Change*, vol. 79, no. 5, pp. 903–918, Jun. 2012, doi: 10.1016/j.techfore.2011.12.001.

[42] B. K. Sovacool, ‘Contestation, contingency, and justice in the Nordic low-carbon energy transition’, *Energy Policy*, vol. 102, pp. 569–582, Mar. 2017, doi: 10.1016/j.enpol.2016.12.045.

[43] S. A. Almohaimeed, S. Suryanarayanan, and P. O’Neill, ‘Reducing carbon dioxide emissions from electricity sector using demand side management’, *Energy Sources, Part A: Recovery, Utilization, and Environmental Effects*, pp. 1–21, May 2021, doi: 10.1080/15567036.2021.1922548.

[44] E. Guelpa and V. Verda, ‘Demand response and other demand side management techniques for district heating: A review’, *Energy*, vol. 219, p. 119440, Mar. 2021, doi: 10.1016/j.energy.2020.119440.

[45] B. Park, J. Dong, B. Liu, and T. Kuruganti, ‘Decarbonizing the grid: Utilizing demand-side flexibility for carbon emission reduction through locational marginal emissions in distribution networks’, *Appl Energy*, vol. 330, p. 120303, Jan. 2023, doi: 10.1016/j.apenergy.2022.120303.

[46] B. Revell, ‘Meat and Milk Consumption 2050: the Potential for Demand-side Solutions to Greenhouse Gas Emissions Reduction’, *EuroChoices*, vol. 14, no. 3, pp. 4–11, Dec. 2015, doi: 10.1111/1746-692X.12103.

[47] S. Xia *et al.*, ‘Creating a low carbon economy through green supply chain management: investigation of willingness-to-pay for green products from a consumer’s perspective’, *International Journal of Logistics Research and Applications*, pp. 1–31, Sep. 2022, doi: 10.1080/13675567.2022.2115988.

[48] Y. Tan, X. Ying, W. Gao, S. Wang, and Z. Liu, ‘Applying an extended theory of planned behavior to predict willingness to pay for green and low-carbon energy transition’, *J Clean Prod*, vol. 387, p. 135893, Feb. 2023, doi: 10.1016/j.jclepro.2023.135893.

[49] Glasgow Financial Alliance for Net Zero, ‘Scaling Transition Finance and Real-economy Decarbonization’, Dec. 2023. Accessed: Dec. 04, 2023. [Online]. Available: https://assets.bbhub.io/company/sites/63/2023/11/Transition-Finance-and-Real-Economy-Decarbonization-December-2023.pdf

[50] L. Bird *et al.*, ‘Wind and solar energy curtailment: A review of international experience’, *Renewable and Sustainable Energy Reviews*, vol. 65, pp. 577–586, Nov. 2016, doi: 10.1016/j.rser.2016.06.082.

[51] P. Moriarty and D. Honnery, ‘Can renewable energy power the future?’, *Energy Policy*, vol. 93, pp. 3–7, Jun. 2016, doi: 10.1016/j.enpol.2016.02.051.

[52] E. Zozmann, L. Göke, M. Kendziorski, C. Rodriguez del Angel, C. von Hirschhausen, and J. Winkler, ‘100% Renewable Energy Scenarios for North America—Spatial Distribution and Network Constraints’, *Energies (Basel)*, vol. 14, no. 3, p. 658, Jan. 2021, doi: 10.3390/en14030658.

[53] H. J. Herzog, ‘Scaling up carbon dioxide capture and storage: From megatons to gigatons’, *Energy Econ*, vol. 33, no. 4, pp. 597–604, Jul. 2011, doi: 10.1016/j.eneco.2010.11.004.

[54] A. Rabiee, A. Keane, and A. Soroudi, ‘Technical barriers for harnessing the green hydrogen: A power system perspective’, *Renew Energy*, vol. 163, pp. 1580–1587, Jan. 2021, doi: 10.1016/j.renene.2020.10.051.

[55] T. Jäger, R. McKenna, and W. Fichtner, ‘The feasible onshore wind energy potential in Baden-Württemberg: A bottom-up methodology considering socio-economic constraints’, *Renew Energy*, vol. 96, pp. 662–675, Oct. 2016, doi: 10.1016/j.renene.2016.05.013.

[56] I. F. A. Vis and E. Ursavas, ‘Assessment approaches to logistics for offshore wind energy installation’, *Sustainable Energy Technologies and Assessments*, vol. 14, pp. 80–91, Apr. 2016, doi: 10.1016/j.seta.2016.02.001.

[57] International Energy Agency, ‘Energy Technology Perspectives 2023’, Jan. 2023.

[58] D. Kampmann, A. Rose, and G. Shrimali, ‘Assessing the Credibility of Climate Transition Plans in the Steel Sector’, Jul. 2023. Accessed: Aug. 16, 2023. [Online]. Available: https://sustainablefinance.ox.ac.uk/wp-content/uploads/2023/07/SSEE-Discussion-Paper-Steel_final_AR.pdf

[59] V. Fthenakis, J. E. Mason, and K. Zweibel, ‘The technical, geographical, and economic feasibility for solar energy to supply the energy needs of the US’, *Energy Policy*, vol. 37, no. 2, pp. 387–399, Feb. 2009, doi: 10.1016/j.enpol.2008.08.011.

[60] S. Ćetković and A. Buzogány, ‘Between markets, politics and path-dependence: Explaining the growth of solar and wind power in six Central and Eastern European countries’, *Energy Policy*, vol. 139, p. 111325, Apr. 2020, doi: 10.1016/j.enpol.2020.111325.

[61] S. Davidsson, L. Grandell, H. Wachtmeister, and M. Höök, ‘Growth curves and sustained commissioning modelling of renewable energy: Investigating resource constraints for wind energy’, *Energy Policy*, vol. 73, pp. 767–776, Oct. 2014, doi: 10.1016/j.enpol.2014.05.003.

[62] R. Kleijn and E. van der Voet, ‘Resource constraints in a hydrogen economy based on renewable energy sources: An exploration’, *Renewable and Sustainable Energy Reviews*, vol. 14, no. 9, pp. 2784–2795, Dec. 2010, doi: 10.1016/j.rser.2010.07.066.

[63] V. V. Klimenko, S. V. Ratner, and A. G. Tereshin, ‘Constraints imposed by key-material resources on renewable energy development’, *Renewable and Sustainable Energy Reviews*, vol. 144, p. 111011, Jul. 2021, doi: 10.1016/j.rser.2021.111011.

[64] V. Moreau, P. Dos Reis, and F. Vuille, ‘Enough Metals? Resource Constraints to Supply a Fully Renewable Energy System’, *Resources*, vol. 8, no. 1, p. 29, Jan. 2019, doi: 10.3390/resources8010029.

[65] International Energy Agency, ‘World Energy Outlook 2023’, Oct. 2023. Accessed: Nov. 27, 2023. [Online]. Available: https://origin.iea.org/reports/world-energy-outlook-2023

[66] IIMA and C. Barrington, ‘OBMS & CARBON NEUTRAL STEELMAKING Whitepaper 3: Future DRI Production & Iron Ore Supply’, May 2022. Accessed: Jun. 04, 2023. [Online]. Available: https://www.metallics.org/assets/files/Public-Area/Decarbonisation/Paper3_DRIProduction.pdf

[67] I. Capellán-Pérez, C. de Castro, and I. Arto, ‘Assessing vulnerabilities and limits in the transition to renewable energies: Land requirements under 100% solar energy scenarios’, *Renewable and Sustainable Energy Reviews*, vol. 77, pp. 760–782, Sep. 2017, doi: 10.1016/j.rser.2017.03.137.

[68] E. Dupont, R. Koppelaar, and H. Jeanmart, ‘Global available wind energy with physical and energy return on investment constraints’, *Appl Energy*, vol. 209, pp. 322–338, Jan. 2018, doi: 10.1016/j.apenergy.2017.09.085.

[69] M. Hajto, Z. Cichocki, M. Bidłasik, J. Borzyszkowski, and A. Kuśmierz, ‘Constraints on Development of Wind Energy in Poland due to Environmental Objectives. Is There Space in Poland for Wind Farm Siting?’, *Environ Manage*, vol. 59, no. 2, pp. 204–217, Feb. 2017, doi: 10.1007/s00267-016-0788-x.

[70] W. Krewitt and J. Nitsch, ‘The potential for electricity generation from on-shore wind energy under the constraints of nature conservation: a case study for two regions in Germany’, *Renew Energy*, vol. 28, no. 10, pp. 1645–1655, Aug. 2003, doi: 10.1016/S0960-1481(03)00008-9.

[71] D. Ryberg, M. Robinius, and D. Stolten, ‘Evaluating Land Eligibility Constraints of Renewable Energy Sources in Europe’, *Energies (Basel)*, vol. 11, no. 5, p. 1246, May 2018, doi: 10.3390/en11051246.

[72] D. Kan, R. Patel, K. Leach, S. Bekker, K. Dawkins, and W. Broer, ‘Biodiversity impact and ecosystem service dependencies: Integration of dependencies using the BFFI and ENCORE’, Sep. 2021. Accessed: Nov. 24, 2023. [Online]. Available: file:///C:/Users/smit0233/Downloads/Dependencies+in+the+BFFI_UNEP_v1.1.pdf

[73] P. Moriarty and D. Honnery, ‘What is the global potential for renewable energy?’, *Renewable and Sustainable Energy Reviews*, vol. 16, no. 1, pp. 244–252, Jan. 2012, doi: 10.1016/j.rser.2011.07.151.

[74] N. Jagger, T. Foxon, and A. Gouldson, ‘Skills constraints and the low carbon transition’, *Climate Policy*, vol. 13, no. 1, pp. 43–57, Jan. 2013, doi: 10.1080/14693062.2012.709079.

[75] J. Brennan and H. Limmer, ‘Sector insights: skills and performance challenges in the energy sector’, Mar. 2015.
